# The Effects of Prenatal Protein Restriction on **β**-Adrenergic Signalling of the Adult Rat Heart during Ischaemia Reperfusion

**DOI:** 10.1155/2012/397389

**Published:** 2012-03-25

**Authors:** Kevin J. P. Ryan, Matthew J. Elmes, Simon C. Langley-Evans

**Affiliations:** Division of Nutritional Sciences, University of Nottingham, Loughborough, Leicestershire LE12 5RD, UK

## Abstract

A maternal low-protein diet (MLP) fed during pregnancy leads to hypertension in adult rat offspring. Hypertension is a major risk factor for ischaemic heart disease. This study examined the capacity of hearts from MLP-exposed offspring to recover from myocardial ischaemia-reperfusion (IR) and related this to cardiac expression of *β*-adrenergic receptors (*β*-AR) and their associated G proteins. Pregnant rats were fed control (CON) or MLP diets (*n* = 12 each group) throughout pregnancy. When aged 6 months, hearts from offspring underwent Langendorff cannulation to assess contractile function during baseline perfusion, 30 min ischemia and 60 min reperfusion. CON male hearts demonstrated impaired recovery in left ventricular pressure (LVP) and *dP*/*dt*_max_ (*P* < 0.01) during reperfusion when compared to MLP male hearts. Maternal diet had no effect on female hearts to recover from IR. MLP males exhibited greater membrane expression of **β**_2_-AR following reperfusion and urinary excretion of noradrenaline and dopamine was lower in MLP and CON female rats versus CON males. In conclusion, the improved cardiac recovery in MLP male offspring following IR was attributed to greater membrane expression of **β**_2_-AR and reduced noradrenaline and dopamine levels. In contrast, females exhibiting both decreased membrane expression of **β**_2_-AR and catecholamine levels were protected from IR injury.

## 1. Introduction

Adult cardiovascular disease is associated with metabolic and physiological aberrations that occur during fetal development. Epidemiological studies provide evidence that intrauterine growth retardation increases the risk of hypertension and cardiovascular disease in adult life [1]. Such findings led to the fetal programming hypothesis that exposure to a suboptimal intrauterine environment can predispose to adult noncommunicable disease [2]. Animal models setup to test the hypothesis demonstrates the onset of cardiovascular pathologies by limiting fetal and neonatal growth through global maternal nutrient restriction [3], protein [4, 5], or micronutrient [6] restriction. The well-established maternal low-protein rat model produces offspring that develop elevated blood pressure which persists throughout adult life [7, 8]. Hypertension is a major independent risk factor for the development of ischemic cardiovascular conditions such as myocardial infarction and stroke [9]. Our laboratory has recently reported that male rats exposed to protein restriction *in utero* showed impaired cardiac contractile recovery to ischemia-reperfusion (IR) injury compared to offspring from control fed dams. Protein restriction had no effect on recovery of the female heart [10]. This sex-specific effect concurs with previous findings in rats in which intrauterine growth restriction as a result of either hypoxia or undernutrition caused cardiac remodelling and impaired recovery to IR in adult male offspring [11].

*β*-adrenergic signalling is key to cardiac contractile function and cardiac recovery following IR. The catecholamines adrenaline and noradrenaline activate myocardial cell surface *β*-adrenergic receptors. *β*_1_ and *β*_2_-AR are the principal *β*-receptor subtypes in the heart making up approximately 70–80% and 30–20% of the *β*-ARs expressed, respectively [12]. Adrenaline stimulates both *β*_1_ and *β*_2_-AR subtypes whereas noradrenaline acts mainly through *β*_1_-AR [13]. *β*-AR activation involves release of the stimulatory G*α*s subunit from the receptor. This stimulates adenylyl cyclase activity and generation of cAMP- and PKA-mediated activation of the contraction-relaxation cycle [12, 14]. These effects are more tightly regulated following *β*_2_-AR activation which acts in an antagonistic fashion by coupling G*α*i leading to the attenuation of cAMP-mediated activation of PKA. Continual *β*-AR activation results in phosphorylation and receptor desensitisation through G protein uncoupling [12]. The receptors then translocate from the plasma membrane into vesicular cytosolic compartments and either targeted for degradation or dephosphorylation and recycled back to the membrane.

The interaction between IR injury and *β*-AR signalling in the heart has been widely investigated and established that myocardial ischaemia is commonly associated with an increase in membrane expression of *β*-ARs and that their overexpression prior to ischaemia increases IR-injury [15–19]. However, the effects of IR on *β*-AR signalling in developmental programming are yet to be explored. This study therefore aims to examine the capacity of isolated adult rat hearts from protein-restricted offspring to recover from IR injury in relation to alterations in shuttling of *β*-AR and G protein signalling between the sarcolemma and cytosolic compartments of the myocardium.

## 2. Methods

### 2.1. Animals

Twenty-four virgin Wistar rat dams (Harlan Ltd, Belton, Leicestershire, UK) weighing approximately 250 g were mated in the animal facilities at the University of Nottingham. The appearance of a semen plug on the cage floor confirmed that mating and rats were allocated either a control (CON) diet (180 g casein/kg, *n* = 12) or a low-protein diet (90 g casein/kg, *n* = 12, MLP), as described previously in detail [7]. The pregnant rats were then fed the isoenergetic semisynthetic diets throughout gestation until birth at 22 days of gestation. All mothers were then transferred to standard laboratory chow diet (B&K Universal Ltd, Hull, UK) and each litter culled to a maximum of 8 pups to minimise variation in the nutrition of the offspring during suckling. At 4 weeks of age, all offspring were weaned onto chow diet, to ensure that litters from control and low-protein fed rat dams only differed in terms of their prenatal nutrition. All experimental procedures were performed in accordance with the Home Office Guidance on the Operation of the Animals (Scientific Procedures) Act, 1986.

### 2.2. The Isolated Heart (Langendorff) Preparation

At 6 months of age, male and female rats from each CON and MLP litter were randomly selected (*n* = 9 or 10), anaesthetised using 3% isofluorane in 2 litres O_2_/min and killed by cervical dislocation. The heart was rapidly excised and cannulated via the aorta to Langendorff perfusion apparatus within 90 seconds (AD Instruments, Oxford, UK) and perfused with Krebs Henseleit buffer (118 mM NaCl, 4.7 mM KCl, 1.2 mM KH_2_PO_4_, 1.2 mM MgSO_4_, 25 mM NaHCO_3_, 11 mM glucose, and 1.25 mM CaCl_2_ (pH 7.4)) bubbled with O_2_-CO_2_ (95 : 5, v/v) in a coronary retrograde fashion. Perfusion pressure was maintained at a constant pressure of 60 mmHg, with perfusate warmed to 37.4°C, and the heart immersed in a water-jacketed temperature-controlled glass chamber set at 37.4°C, therefore ensuring normothermia throughout the perfusion protocol. Contractile function was monitored by the careful insertion of a saline filled latex balloon (Linton Instruments) into the left ventricle which was adjusted to an end diastolic pressure of 5–10 mmHg. Left ventricular and perfusion pressure were continuously monitored through precalibrated physiological pressure transducers (Senso-Nor 844, AD Instruments). Data recording was not started until all variables were stable (15–30 min) after which the following 30 min were defined as baseline. Ischemia was then induced by switching off the coronary perfusion apparatus for a 30-minute period. Coronary perfusion was then reinstated for a further 60-minute period to assess cardiac responses during reperfusion. Data for left ventricular developed pressure (LVP), heart rate (HR) and left ventricular first derivative (*dP*/*dt*_max⁡_) were collected and processed using the Powerlab Data Acquisition System (AD) Instruments. To determine the effect of IR on myocardial expression of *β*-AR, hearts from CON or MLP offspring that underwent either (i) 30 min baseline perfusion alone (*n* = 9-10), (ii) 30 min baseline perfusion followed by 30 min ischemia (*n* = 9-10) or (iii) 30 min baseline perfusion followed by 30 min ischemia and 60 min reperfusion (*n* = 9-10) on the Langendorff apparatus were collected and snap frozen for western blot procedures.

### 2.3. Western Blot Analysis

Hearts collected for western blot analysis from the Langendorff study were used to prepare cardiac membrane and cytosolic fractionated samples as described by Fernandez-Twinn and colleagues [20]. Frozen whole heart tissue was ground to a powder in liquid nitrogen and homogenised briefly for 30 s in ice cold buffer containing 5 mM Tris pH 7.4, 2 mM EDTA, and protease inhibitor cocktail (Calbiochem). Homogenates underwent centrifugation at 1000× g for 15 min at 4°C to pellet nuclear material. The supernatant fraction was then spun at 100,000× g for 1 h 20 min at 4°C in an ultracentrifuge to generate a pellet and supernatant which corresponded to the plasma membrane and cytosolic fractions, respectively. The pellet was then resuspended in lysis buffer (50 mM HEPES pH 8, 150 mM NaCl, 1% Triton-X-100, 1 mM Na_3_VO_4_, 30 mM NaF, 10 mM Na_4_P_2_O_7_, 10 mM EDTA, and protease inhibitor cocktail).

Protein concentrations of the supernatant and resuspended pellet fractions were determined using the Bio-Rad protein assay system (Bio-Rad, Hemel Hempstead, UK) according to the manufacturer's instructions. Samples were standardised to a concentration of 3 mg/mL with Laemmli's sample buffer (62.5 mM Tris pH 6.8, 2% SDS, 10% glycerol, 0.02% bromophenol blue, 150 mM dithiothreitol) and boiled for 3 min before equal protein quantities of each sample were separated by SDS PAGE. Proteins were transferred to nitrocellulose membrane (Hybond-C extra, Amersham Bioscience) for probing with antibodies to *β*_1_-AR (Affinity Bioreagents; rabbit polyclonal raised against a synthetic peptide corresponding to residues 394–408 of mouse/rat *β*_1_-AR), *β*_2_-AR (Abcam; rabbit polyclonal raised against against residues 1–100 of human *β*_2_-AR), G stimulatory protein (Santa Cruz G*α*s (K-20): sc-823), and G inhibitory protein (Santa Cruz G*α*i-3 (C10): sc262). Membranes were incubated in blocking solution (5% dried skimmed milk in TBS with 1% Tween 20) prior to incubation with primary antibodies. Horseradish peroxidise secondary antibody conjugated to rabbit IgG was used at a working concentration of 1 : 5000 (GE healthcare, Amersham, UK). Bands were developed on high performance chemiluminescence film (Hyperfilm ECL, Amersham) using ECL Plus reagent (GE healthcare, Amersham, UK). Densitometric analysis of band intensity across gels was performed using a Biorad Gel Doc XR imaging system and Quantity One 1D analysis software. Three replicates of a pooled extract were included on every gel and used as a standard to facilitate gel-to-gel comparisons.

### 2.4. Urine Collection and Catecholamine Assays

Rats at 5 weeks, 10 weeks, and 6 months of age were housed in metabolic cages for 24 hours and urine collected as previously described [21]. Urine concentrations of adrenaline, noradrenaline, and dopamine were determined using the DLD Diagnostika catecholamine ELISA kit (DLD Diagnostika GmbH, Hamburg, Germany), following the manufacturer's instructions.

### 2.5. Statistical Analysis

All results are presented as mean values with their standard errors. All data were analysed for homogeneity of variance, and if heterogeneous transformed by square root or logarithmic conversion. For the analyses, ischemia-reperfusion data were split into three distinct time bins: (1) 30 min baseline, (2) recovery 1 (0–30 min of reperfusion), and (3) recovery 2 (31–60 min of reperfusion). The effects of maternal diet on the recovery in LVP, HR, and *dP*/*dt*_max⁡_ relative to baseline were analysed using a one-way repeated measure ANOVA. Western blot data were analysed by three-way ANOVA considering maternal diet, sex, and ischemia-reperfusion treatment as fixed factors. ELISA data were analysed by three-way ANOVA considering maternal diet, sex, and age as fixed factors. Measurements from related animals from the same litter were grouped in all ANOVA analyses and assigned as a random factor. All statistical analyses were performed using SPSS version 18. In all analyses, *P* < 0.05 was considered statistically significant.

## 3. Results

### 3.1. Effect of Maternal Diet on Functional Recovery of Isolated Hearts to IR Injury

The effect of maternal diet on the recovery of contractile function relative to baseline in the first (R1) and second (R2) 30 min periods of reperfusion was measured in Langendorff-perfused hearts from male and female rats. Maternal diet had no effect on LVP, HR, or *dP*/*dt*_max⁡_ baseline indices in either sex (Table 1). Similarly, there was no significant effect of maternal diet on the recovery of female indices of cardiac function (LVP, *dP*/*dt*_max⁡_,  HR) to ischemia-reperfusion insult (Table 1). However, significant differences in the capacity of male hearts to recover from ischemia-reperfusion injury were observed between CON- and MLP-exposed offspring. Interestingly the recovery of LVP in MLP offspring during R1 and R2 phases of reperfusion was 2-fold higher than the recovery seen in CON males (*P* < 0.01) (Table 1). A similar result was observed with *dP*/*dt*_max⁡_ where MLP males exhibited almost a 2-fold greater recovery during the R1 phase (*P* < 0.05) and a trend (*P* = 0.06) for an improved recovery during the R2 phase in comparison to CON hearts (Table 1). In contrast, there was no significant effect of maternal diet on recovery of heart rate (Table 1).

### 3.2. *β* Adrenergic Signalling Response to IR Injury in the Isolated Rat Heart

**Figure 1:**
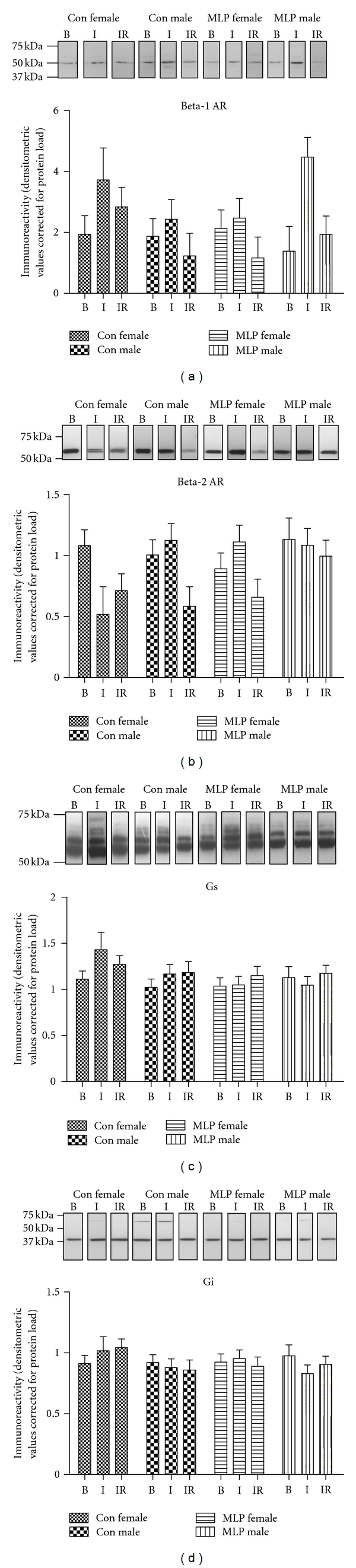
*β*-adrenergic receptor (AR) isoforms, Gs, and Gi protein expression in cardiac membranes from CON and MLP Langendorff perfused rat hearts at baseline and following ischemia and ischemia reperfusion. Cardiac membrane expression of *β*_1_-AR (a), *β*_2_-AR, (b) Gs (c), and Gi (d) was determined by western blot analysis. Data were expressed as mean ± SEM values for isolated hearts exposed to either baseline (B) perfusion, 30 min ischemia (I), or 30 min ischemia followed by 60 min reperfusion (IR) from Con Female, Con Male, or protein-restricted (MLP) MLP Female or MLP Male rats. Data were analysed by three-way ANOVA. *β*_1_-AR showed an interaction effect between maternal diet and sex (*P* < 0.05) and an effect of ischemia reperfusion treatment (*P* < 0.01). *β*_2_-AR expression showed an interaction effect of maternal diet, sex, and ischemia reperfusion treatment (*P* < 0.05).

**Figure 2:**
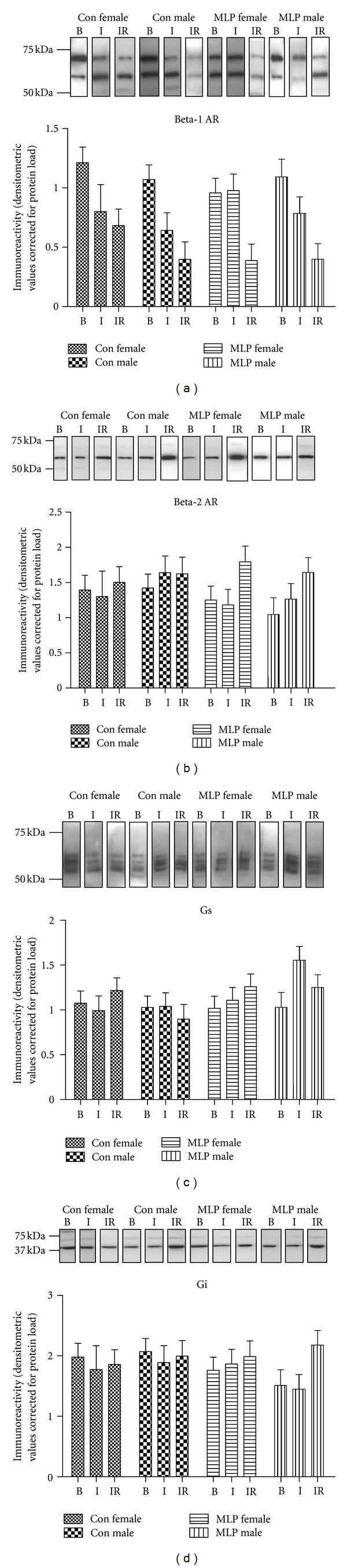
*β*-adrenergic receptor (AR) isoforms, Gs, and Gi protein expression in cardiac cytosolic fractions from CON and MLP Langendorff perfused rat hearts at baseline and following ischemia and ischemia reperfusion. Cardiac cytosolic expression of *β*_1_-AR (a), *β*_2_-AR (b), Gs (c), and Gi (d) was determined by western blot analysis. Data were expressed as mean ± SEM values for isolated hearts exposed to either baseline (B) perfusion, 30 min ischemia (I) or 30 min ischemia followed by 60 min reperfusion (IR) from Con Female, Con Male, or protein-restricted (MLP) MLP Female or MLP Male rats. Data were analysed by three-way ANOVA. *β*_1_-AR and *β*_2_-AR showed effects of ischemia reperfusion treatment (*P* < 0.001).

*β* adrenergic signalling is a central mechanism involved in the maintenance of cardiac inotropic and chronotropic function and has been shown to play a key role in recovery of the heart from IR. Accordingly, we quantified levels of the two main *β*-AR isoforms, *β*_1_-AR and *β*_2_-AR, as well as levels of the stimulatory and inhibitory G proteins G*α*s and G*α*i, respectively, in isolated hearts exposed either to baseline perfusion (B), baseline perfusion plus ischemia (I), or baseline perfusion plus ischemia plus reperfusion (IR). Expression was measured in both membrane (Figure 1) and cytosolic (Figure 2) fractions prepared from each heart. Baseline membrane or cystolic expression of all proteins was not significantly altered by gender or maternal diet; however differential levels of expression were observed following ischemia and ischemia-reperfusion.

*β*_1_-AR levels at the membrane increased following ischemia and returned to baseline levels by the end of 60 min reperfusion (Table 1; *P* < 0.05). This correlated with a decline in *β*_1_-AR in the cytosol by the end of the ischemic period which had declined further by the end of reperfusion (Figure 2(a); *P* < 0.001). A significant interaction between diet and sex (*P* < 0.05) was also observed for membrane expression of *β*_1_-AR indicating that the overall level of receptor expression in females was lower in the MLP group when compared to CON, an effect not observed in males.

Cardiac membrane expression of *β*_2_-AR revealed a significant interaction between maternal diet, sex, and ischemia-reperfusion treatment (Figure 1(b); *P* < 0.05). *β*_2_-AR decreased in CON females following ischemia which was in complete contrast to all other groups where receptor expression levels were unchanged from baseline levels. Following reperfusion, *β*_2_-AR expression in CON females remained unchanged from levels observed after ischemia. Hearts from CON males showed decreased membrane expression of *β*_2_-AR after reperfusion similar to the decreased expression observed in CON female hearts following ischemia (Figure 1(b)). However, hearts from MLP females and males maintained baseline levels of *β*_2_-AR expression throughout ischemia and reperfusion.

*β*_2_-AR expression in cardiac cytosolic fractions was found to increase after ischemia and increased further following reperfusion (Figure 2(b); *P* < 0.001) which was in direct contrast to *β*_1_-AR cytosolic expression. These findings suggest that ischemia-reperfusion initiates a differential response in membrane trafficking of *β*_1_-AR and *β*_2_-AR from the cytosol to the cardiac membrane. Despite changes in *β*-AR isoform within the cardiac membrane and cytosol, no overt changes were observed in the levels of Gs and Gi (Figure 1(d) and Figure 2(d)) proteins within either of these cellular locations.

### 3.3. Urinary Catecholamine Levels in the Rat at 5 Weeks, 10 Weeks, and 6 Months of Age

Concentrations of the catecholamines adrenaline, noradrenaline, and dopamine were measured in the urine of rats at 5 weeks, 10 weeks, and 6 months of age. No significant differences were observed in the concentration of adrenaline at each stage of development (Table 2). Urine levels of noradrenaline however were significantly affected by maternal diet where greater levels were observed in CON compared to MLP rats (Table 2; *P* < 0.02). In addition, a significant effect of age was observed where noradrenaline concentrations were significantly higher at 5 and 10 weeks of age respectively (*P* < 0.02). An interaction between age and sex was also evident (*P* < 0.01) where urine concentrations of noradrenaline in females were highest at 5 weeks of age with a concentration of 15 ng/mL before dropping to 8 ng/mL at 10 weeks of age, and remaining at this level when aged 6 months. For males, noradrenaline concentration at 10 weeks of age had risen from 10 ng/mL observed at 5 weeks to 14 ng/mL before dropping by 60% to 8 ng/mL at 6 months of age.

Dopamine concentrations were significantly reduced by an MLP diet (Table 2; *P* < 0.01), and an interaction between sex and age was also observed (Table 2; *P* < 0.04). Female rats had a significantly reduced urinary excretion of dopamine at 6 months of age compared to both 5 and 10 weeks of age. In contrast, urinary excretion of dopamine in male rats increased significantly with age. At 6 months of dopamine concentrations were significantly higher than the level determined at 5 and 10 weeks of age.

## 4. Discussion

The key finding from the present study is that hearts from MLP male offspring demonstrated improved recovery in cardiac function following IR when compared to CON males, whereas IR recovery in females was unaffected by maternal diet. This study verifies that sex is an important factor in the capacity of the heart to recover from IR injury [10, 15, 22–24]. This contrasts starkly with our previous finding where CON male rats demonstrated improved contractile recovery to IR compared to CON females or MLP rats of either sex [10]. This discrepancy may be explained through experimental differences. In the initial study, rats were restraint for intraperitoneal injections and subject to restraint stress. This procedure was absent in the present study. Introduction of restraint prior to Langendorff perfusion may have conditioned the CON male rat hearts' response to stress and enhanced their capacity to recover from IR injury. Stressors, such as hyperthermia, hypo- and hyperoxia, and dexamethasone treatment, have been observed to precondition and protect hearts from subsequent ischemic injury [25–28]. If true, hearts from either CON females or MLP of either sex were not receptive to preconditioning. This is consistent with the cardioprotective effects of ischemic preconditioning only observed in male and not female mouse hearts [29]. Interestingly, there was no significant difference in cardiac recovery following ischemia in MLP rats between the present and previous study. This suggests that maternal protein restriction may block the positive effects of preconditioning. MLP rats consistently develop elevated blood pressure which persists throughout adulthood and may prevent cardiac preconditioning in these animals. Indeed evidence suggests that hypertensive individuals hearts are not susceptible to preconditioning [30]. Previous work has shown that protein restriction programmes changes in the hypothalamic-pituitary-adrenal axis, which is likey to have a major impact on the response of MLP rats to stress [31]. 

To investigate how cardiac function during IR relates to *β*-AR signaling, we determined protein expression of the *β*-AR isoforms, and the Gs and Gi proteins in baseline-perfused ischemia or ischemia-reperfused hearts. *β*_1_-AR expression was associated with increased expression within the plasma membrane after ischemia that returned to baseline levels following reperfusion. This suggests that the *β*_1_-AR expression is upregulated in the membrane in response to ischemia and declines throughout reperfusion. This agrees with Strasser et al. [19], who identified that sarcolemmal *β*_1_-AR levels increase following 50 min ischemia that was associated with decreased adenylyl cyclase activity. More recently, *β*_1_-AR protein expression in isolated rat hearts has also been demonstrated to increase at the plasma membrane following 30 min ischemia but with no change in adenylyl cyclase activity [32]. Further agreement with this study is that *β*_1_-AR density at the plasma membrane decreases following 60 min reperfusion compared to levels after ischemia which was associated with a doubling in adenylyl cyclase activity [32]. It is important to concede that the changes in *β*_1_-AR expression following ischemia and reperfusion in the present study may not be reflective of downstream changes at the level of adenylyl cyclase.

Protein expression of *β*_2_-AR in the cardiac membrane decreased following ischaemia in CON females and following reperfusion in CON males and MLP females. These findings demonstrate subtle sex-specific responses in downregulation of *β*_2_-AR at the cardiac membrane to ischaemia and reperfusion. To our knowledge, this is the first time sex-specific effects of IR on *β*_2_-AR abundance have been reported. A previous study focussing solely on male hearts reported that *β*_2_-AR density at the plasma membrane was unaffected following 30 min ischemia but decreased with 60 min reperfusion, thus agreeing with the present findings in CON males [32]. In contrast *β*_2_-AR protein expression in the cardiac membrane of MLP males did not change throughout ischemia and reperfusion from baseline levels. Consequently, *β*_2_-AR levels were higher in MLP males compared to all other treatment groups following reperfusion.

All these changes in BAR expression at the membrane occurred against a background where neither Gs nor Gi expression levels changed, suggesting that *β*-AR density rather than G protein content at the cardiac membrane is the rate limiting component mediating cardiac contractility. Alterations in expression of *β*-AR isoforms through transgenic mouse models show profound effects on haemodynamic function of the heart [15, 33–35]. Recent work has focussed on the role of *β*_2_ rather than the *β*_1_-AR activation in cardiac pathology due to its specific capacity to enhance Ca^2+^ homeostasis and protection against apoptosis [35–38]. However, transgenic mouse models have shown that these therapeutic effects cannot be recapitulated by simply overexpressing *β*_2_-AR of the heart [16, 34, 35]. Despite increased inotropic and chronotropic activity, cardiac *β*_2_-AR overexpression led to progressive cardiac hypertrophy and delayed onset cardiomyopathy. Moreover, *β*_2_-AR overexpression exacerbated ischemic injury but in the hearts of male mice only [15]. Female mice overexpressing *β*_2_-AR were protected from damage following IR and linked to the female sex hormone oestrogen acting through endothelial nitric oxide synthase to prevent Ca^2+^ dysregulation in the tissue [15]. Such findings suggest that the contractile function of the female heart is less sensitive to *β*_2_-AR expression changes. This may explain why cardiac *β*-AR expression levels observed between CON and MLP groups in the present study resulted in differences in haemodynamic parameters in male rats only.

The cardiac pathology observed in transgenic mouse models is possibly an artefact of such high *β*_2_-AR overexpression and does not represent a moderate increase in *β*_2_-AR expression [34]. Comparisons of *β*_2_-AR overexpression have shown that 100–360-fold increases in *β*_2_-AR expression resulted in the onset of cardiomyopathy, whereas a moderate 60-fold increase had no overt pathological consequence [34]. Indeed recent studies have presented evidence supporting the putative protective role of *β*_2_-AR signalling against cardiac pathology [37–39]. Long-term *β*_2_-AR agonist treatment in conjunction with a *β*_1_-AR blocker was found to improve survival and contractility in a rat model of dilated cardiomyopathy [36, 37]. Furthermore treatment with the *β*_2_-AR agonist clenbuterol alone was sufficient to exert a cardioprotective effect in rat hearts through a reduction in myocardial apoptosis and oxidative stress and improving diastolic function following IR injury [38]. This cardioprotective effect of *β*_2_-AR activation is thought to occur via stimulation of Gi rather than Gs-mediated signalling mechanisms which enable Ca^2+^ regulation to be maintained and apoptosis to be inhibited.

Our key finding is that MLP male hearts do not downregulate *β*_2_-AR protein expression within the cardiac membrane following IR, which is in complete contrast to CON males. We propose that downregulation of *β*_2_-AR in response to IR is the cause of impaired contractile recovery in CON male hearts. Cardiac membrane expression of *β*_2_-AR also decreased after reperfusion in CON and MLP female hearts to a level comparable to CON males but despite this decrease showed no impairment in contractile recovery following ischemia. This may result from the protective role of oestrogen in maintaining a favourable Ca^2+^ microenvironment within female hearts during reperfusion that limits tissue damage and counteracts any changes in *β*-adrenergic signalling activity that would otherwise impair contractile recovery after ischemia. Measurements of urinary catecholamine levels indicate that by 6 months of age there is no overt difference in either adrenaline or noradrenaline levels in these animals. However, we did observe an increase in dopamine levels in males compared to female rats at 6 months of age. It is not clear how this difference affects cardiac function of these hearts. Dopamine is routinely administered as a therapy in heart failure to improve cardiac function [39]. This is thought to occur primarily through activation of *β*-ARs in the heart. We did not see any evidence to indicate that this sex-specific difference in dopamine had any impact on basal levels of expression of any of the *β*-adrenergic signalling components measured in this study or on baseline activity of the hearts prior to ischemic reperfusion. Further work is required to explore other potential receptor targets of dopamine which may alter cardiac function in response to ischemia reperfusion.

Urine excretion of noradrenaline and dopamine was significantly lower in MLP compared to CON animals and may offset the ischaemic damage observed in CON males. Evidence to support this is the fact that depletion of catecholamines by surgical denervation causes a redistribution of *β*-ARs, decreasing *β*_1_ and increasing the *β*_2_ subtype although total *β*-AR content remained the same [40]. Furthermore, suppression of noradrenaline turnover in the rat heart protects against myocardial IR injury [41].

## 5. Conclusion

We propose that the contractile dysfunction in male CON hearts following IR occurs as a result of increased catecholamine production and decreased *β*_2_-AR expression at the cardiac membrane. Likewise, protein expression of *β*_2_-AR at the cardiac membrane decreased in response to IR in female CON and MLP hearts. However they displayed an improved cardiac recovery compared to CON males suggesting specific protective mechanisms in female hearts that counteract alterations in *β*-adrenergic receptor expression. Our findings indicate that in male offspring prenatal protein restriction maintains cardiac membrane expression of *β*_2_-AR during IR which improves cardiac contractile recovery. In contrast, female hearts were resistant to these effects suggesting subtle sex differences in the molecular mechanisms controlling cardiac membrane expression of *β*-AR in response to IR.

## Figures and Tables

**Table 1:** Effect of maternal protein restriction on the cardiac recovery of male and female rat hearts during ischemia reperfusion. Measures of cardiac function include left ventricular pressure (LVP), the left ventricular first derivative (*dP*/*dt*_max⁡_), and heart rate (HR). These parameters were determined at baseline and during 60 min reperfusion following 30 min ischemia. Data are displayed as mean ± SEM values. Recovery 1 and 2 denote 0–30 and 31–60 min reperfusion periods, respectively.

	Males					Females				
	CON		MLP		*P*-value	CON		MLP		*P* value
	Mean	SE	Mean	SE		Mean	SE	Mean	SE	
LVP (mmHg)										
Baseline	44.67	3.0	50.18	5.3	0.38	43.74	4.1	50.57	5.1	0.31
Recovery 1	5.81	1.0	11.71	1.6	**0.01**	10.83	2.0	10.83	2.3	1.00
Recovery 2	7.25	1.7	15.33	3.3	**0.04**	11.31	2.3	13.24	2.6	0.58
Recovery 1 (% baseline)	13.8	3.3	23.31	4.0	0.09	28.41	6.3	25.16	6.6	0.88
Recovery 2 (% baseline)	18.3	5.3	33.24	8.0	0.16	29.33	6.6	31.11	7.2	0.87
HR (beats per min)										
Baseline	302.42	21.3	290.9	7.0	0.61	331.76	18.8	298.23	19.7	0.23
Recovery 1	112.16	26.5	105	24.4	0.85	129.53	25.1	183.46	26.9	0.16
Recovery 2	195.42	19.3	169.54	28.2	0.47	179.41	31.5	255.36	38.0	0.14
Recovery 1 (% baseline)	30.6	7.1	43.26	6.9	0.50	37.89	7.9	60.22	8.8	0.08
Recovery 2 (% baseline)	65.1	7.1	59.45	9.8	0.66	53.65	10.9	85.03	12.2	0.07
*dP/dt* (mmHg/s)										
Baseline	1186.01	101.9	1398.09	135.5	0.23	1252.42	106.5	1402.94	133.8	0.39
Recovery 1	381.59	32.9	625.55	78.1	**0.01**	515.82	66.5	530.37	53.4	0.87
Recovery 2	409.39	52.1	599.45	79.1	0.06	570.02	84.2	544.59	51.1	0.81
Recovery 1 (% baseline)	31.52	4.1	47.34	7.2	0.08	42.29	5.4	40.35	5.2	0.54
Recovery 2 (% baseline)	35.43	4.7	43.76	6.8	0.33	45.51	5.4	42.78	5.0	0.33

**Table 2:** Catecholamine concentration levels in the urine of rats at 5 weeks, 10 weeks and 6 months of age. Urine was collected over a 24 h period and the concentration (ng/mL) of adrenaline, noradrenaline and dopamine were measured by ELISA. Adrenaline and dopamine were log transformed and Noradrenaline underwent square root transformation to produce homogeneous data. The transformed data was then analysed by three-way ANOVA and expressed as mean and SEM with corresponding  *P*  values for each analysis.

		Adrenaline (ng/mL)		Noradrenaline (ng/mL)		Dopamine (ng/mL)	
		Mean	SEM	Mean	SEM	Mean	SEM
CON Female	5 weeks	7.72	3.23	249.19	57.05	316.14	69.9
	10 weeks	23.01	4.27	128.28	75.48	294.8	83.17
	6 months	18.96	4.27	151.38	61.63	113.37	43.2

MLP Female	5 weeks	6.88	3.82	240.61	53.37	158.64	55.66
	10 weeks	14.40	3.49	51.71	75.48	107.61	29.13
	6 months	14.57	3.02	40.62	67.51	103.9	24.4

CON Male	5 weeks	6.94	3.02	177.08	57.05	116.99	14.35
	10 weeks	11.17	4.93	374.06	61.63	284.9	56.35
	6 months	12.63	3.49	59.58	75.48	276.2	72.5

MLP Male	5 weeks	8.59	4.27	82.80	61.63	163.98	61.4
	10 weeks	8.31	3.49	147.51	67.51	133.11	35.5
	6 months	8.22	3.23	98.86	61.63	148.1	56.5

ANOVA		*P*-values		*P*-values		*P*-values	

Diet		0.117		**0.016**		**0.010**	
Sex		0.115		0.906		0.559	
Age		0.072		**0.022**		0.400	
Diet × Sex		0.662		0.842		0.880	
Diet × Age		0.171		0.623		0.353	
Sex × Age		0.438		**0.011**		**0.038**	
Diet × Sex × Age		0.768		0.105		0.222	
